# Modifiable and Non-Modifiable Predictors of Exercise Capacity in Stroke Survivors: A Systematic Review

**DOI:** 10.3390/healthcare14030382

**Published:** 2026-02-03

**Authors:** Klaske van Kammen, Lotte A. J. Verkuijlen, Ana B. Nasser, Rienk Dekker, Leonie A. Krops, Bregje L. Seves

**Affiliations:** 1 Department of Rehabilitation Medicine, University Medical Center Groningen, University of Groningen, 9713 GZ Groningen, The Netherlandsnasser.anabeatriz@gmail.com (A.B.N.);; 2 Department of Human Movement Sciences, University Medical Center Groningen, University of Groningen, 9713 GZ Groningen, The Netherlands

**Keywords:** stroke, VO_2_peak, systematic review, fatigue, personalized care, cardiorespiratory fitness, rehabilitation treatment, training intensity, lower limb strength, body composition

## Abstract

**Highlights:**

**What are the main findings?**

- Modifiable factors—such as body composition (e.g., BMI), lower limb strength, cardiorespiratory fitness (e.g., baseline VO_2_peak), training intensity, and fatigue—significantly predict exercise capacity in stroke survivors in separate prediction models.
- Non-modifiable factors, including age, diabetes, and stroke severity, also significantly predict exercise capacity in separate prediction models.

**What are the implications of the main findings?**

- Rehabilitation strategies should focus on improving modifiable factors to enhance exercise capacity.
- Incorporating non-modifiable factors into baseline assessments can support more personalized and effective rehabilitation planning.

**Abstract:**

Background: This systematic review aims to identify modifiable and non-modifiable predictors of exercise capacity (VO_2_peak level or change) in stroke survivors. These insights may further optimize rehabilitation treatment and improve long-term health outcomes. Methods: PubMed (PubMed.gov), EMBASE (Elsevier), CINAHL (EBSCO), and Web of Science (Clarivate) were searched (last search on 7 October 2025). Inclusion criteria were: (1) adults (>18 years) who survived a stroke (ischemic and hemorrhagic), (2) outcome was a measurement of maximum exercise capacity (VO_2_peak) measured with CPET (or equivalent), (3) predictors of exercise capacity were measured (e.g., personal factors, disease-related factors, components of rehabilitation), (4) predictors of exercise capacity were analyzed in multivariate regression models, (5) primary research, and (6) full-text available. During the data extraction phase, predictors were categorized into modifiable and non-modifiable predictors. Risk of bias was assessed with the McMaster Critical Review Form for Quantitative Studies. Results: Of 919 unique articles, seventeen were included. Modifiable factors such as BMI (4/8 articles) and fat mass (1/1), lower limb strength (3/3), cardiorespiratory fitness (e.g., baseline VO_2_peak (2/4)), training intensity (2/2) and perceived fatigue (1/1) significantly predicted VO_2_peak (level or change). Significant non-modifiable predictors were age (3/11), sex (1/8), diabetes (1/2), and stroke-specific (4/8) factors. Conclusions: This systematic review highlights the significant role of modifiable and non-modifiable predictors in optimizing exercise capacity (VO_2_peak) for stroke survivors. In addition, considering modifiable and non-modifiable predictors allows for more personalized treatment planning. The findings can guide healthcare professionals in tailoring rehabilitation programs, though further research is needed to develop a comprehensive prediction model.

## 1. Introduction

Annually, the World Stroke Organization reports over 12.2 million new stroke events worldwide, which equals one stroke every three seconds [1]. This makes stroke one of the leading causes of disability and a major public health concern [1]. In 2022, the annual cost of stroke was estimated at 721 billion US dollars, representing 0.66% of the Gross World Product [1]. Stroke survivors often experience impairments, including hemiparesis, sensory deficits, dysarthria, diplopia, and facial droop [2]. Moreover, stroke survivors exhibit significantly reduced exercise capacity compared to age-matched healthy individuals, which poses additional challenges to the rehabilitation process [3,4] and has a major impact on a person’s ability to perform activities of daily living [5]. In addition, reduced exercise capacity is strongly linked to increased cardiovascular morbidity and mortality, and stroke survivors already face an elevated risk of recurrent cardiovascular events [6,7]. Therefore, interventions that effectively improve exercise capacity may contribute to lowering long-term cardiovascular risk. Furthermore, meta-analyses have shown that enhanced exercise capacity through diverse exercise interventions can lead to small to moderate improvements in quality of life [8,9]. This underscores the need for efficient and effective stroke rehabilitation programs that improve exercise capacity, aiming to enhance recovery and, consequently, participation and ultimately reducing societal costs [1]. A crucial step towards achieving this goal is the identification of predictors of exercise capacity in stroke survivors. Understanding these predictors can guide healthcare professionals to design, improve and tailor rehabilitation programs [10].

Cardiopulmonary exercise testing (CPET) is considered the gold standard for measuring peak exercise capacity (VO_2_peak) [11]. VO_2_peak is defined as the highest rate at which oxygen can be absorbed and utilized by the body during intense exercise [12]. Within 0 to 30 days after stroke, the VO_2_peak decreases on average to 10–17 mL/kg/min [13,14], which is only 27–87% of the average VO_2_peak in age- and sex-matched healthy adults [15]. Importantly, low VO_2_peak may not only be a consequence of stroke-related impairments but also represent a well-established risk factor for stroke itself [16]; both mechanisms likely contribute to the markedly reduced VO_2_peak observed in stroke populations. Even after six months, in the chronic phase of stroke, levels of exercise capacity are still reduced to 25% to 45% of the expected value for healthy people with similar characteristics [17].

An important aspect in enhancing exercise capacity of stroke survivors is the implementation of aerobic exercise training in rehabilitation programs. Aerobic exercise training in stroke survivors during rehabilitation was proven effective for increasing VO_2_peak [18,19,20]. Beyond its impact on aerobic capacity, aerobic exercise training contributes to enhanced balance, gait speed, walking ability, cognitive performance, and quality of life [20,21,22,23].

Even though exercise capacity in stroke survivors can be improved with aerobic exercise, considerable inter-individual variability in response has been reported [3]. Potential factors contributing to the variability in response to aerobic exercise training include age, baseline aerobic capacity, and stroke-specific factors such as severity and location [24]. Identifying these different personal and physiologic components can provide valuable insight into the factors that predict the level of exercise capacity in stroke survivors. Categorizing predictors into modifiable predictors (e.g., physiological factors) and non-modifiable predictors (e.g., demographic factors) can help to identify which factors can be targeted through interventions. This approach allows the optimization of personalized rehabilitation programs aiming to improve exercise capacity in stroke survivors.

To our knowledge, no previous systematic review has summarized modifiable and non-modifiable predictors of exercise capacity in stroke survivors. Therefore, this systematic review aims to identify modifiable and non-modifiable factors that predict peak exercise capacity outcome (VO_2_peak) in stroke survivors, either at baseline or after training. The findings of the current study might have the clinical implication to provide evidence-based recommendations that guide healthcare professionals in optimizing and tailoring stroke rehabilitation programs in order to improve long-term health outcomes.

## 2. Methods

This systematic review was conducted according to the Preferred Reporting Items for Systematic Review and Meta-Analyses (PRISMA) [25]. The review protocol was pre-registered in the International Prospective Register of Systematic Reviews (PROSPERO: CRD42024534784).

### 2.1. Search Strategy

The databases PubMed, EMBASE, CINAHL, and Web of Science were searched (final initial search: 13 October 2023; search update: 7 October 2025) using terms related to stroke, exercise capacity, and predictors, aligned with the PICO framework [26]. The earliest publication dates included in the searches were 1989 for PubMed, 1974 for Embase, 1997 for CINAHL, and 1964 for Web of Science. The complete search is described in the Supplementary Materials.

### 2.2. Article Selection

The PICO framework was used to formulate the eligibility criteria [26]. The following inclusion criteria were applied: (1) adults (>18 years) who survived a stroke (ischemic or hemorrhagic), (2) outcome had to be a measurement of exercise capacity (VO_2_peak) measured with CPET (or equivalent) at any time point, (3) predictors were measured (e.g., personal factors, disease-related factors, components of rehabilitation), (4) predictors were analyzed in multivariate regression models intended to predict VO_2_peak (level or change), and (5) full-text available. Articles were excluded when they met at least one of the following criteria: (1) article not in humans and (2) article not in English. No exclusion criteria based on study design were applied to maximize comprehensiveness and ensure inclusion of all relevant evidence.

Measuring exercise capacity with CPET is considered the gold standard [11], and this review primarily focuses on VO_2_peak as the outcome measure to quantify aerobic capacity in stroke survivors. VO_2_peak refers to the highest oxygen consumption achieved during an exercise test, regardless of reason for test termination. In the literature, VO_2_max is also an often-reported outcome, which represents the maximum level of oxygen consumption attainable. However, in patient populations, like stroke survivors, VO_2_max may not be attainable due to factors such as balance issues or pain [27]. Therefore, VO_2_peak is considered the suitable outcome for the current review. For consistency across studies, VO_2_max is considered equivalent to VO_2_peak and is therefore rephrased to VO_2_peak throughout this review.

Two assessors (L.A.J.V. and A.B.N. for the initial search, K.v.K. and B.L.S. for the search update) independently screened the titles and abstracts simultaneously based on the inclusion criteria. Disagreements following title and abstract screening were resolved through a consensus meeting between the assessors. Subsequently, the same assessors completed full-text screening for inclusion, followed by a second consensus meeting. Any unresolved disagreements after title/abstract screening or full-text screening were resolved in a consensus meeting involving a third assessor (B.L.S. for the initial search and L.A.K. for the search update). Cohen’s Kappa and % agreement were calculated for title/abstract screening and full-text screening to determine interrater agreement before the consensus meeting(s). Cohen’s Kappa was interpreted as follows: <0.40 indicated slight to fair agreement, 0.41–0.60 indicated moderate agreement, 0.61–0.80 indicated substantial agreement, and >0.8 indicated almost perfect agreement [28].

### 2.3. Data Extraction

The following data were extracted from the included articles: name of the first author; year of publication; study design; number of participants; sex; mean age; measures of exercise capacity and time point of this measurement; intervention; time since stroke onset; stroke severity; stroke type; stroke localization; predictors of exercise capacity; and details of the final predictions models: dependent variable, model type, regression coefficient/unstandardized beta, standard error (SE), standardized beta, 95% confidence interval and *p*-values of the significant predictors in all multivariate models. Predictors were categorized as modifiable or non-modifiable based on predefined definitions as well as clinical relevance as discussed with the research team. L.A.J.V. and B.L.S. independently classified each predictor. Modifiable predictors were defined as factors that can be influenced through rehabilitation, exercise training, lifestyle changes, or medical management. Predictors that may be partially modifiable in some contexts (e.g., lean mass, habitual physical activity) were also included in this category to maintain a clear two-category framework. Non-modifiable predictors were defined as fixed characteristics such as age, sex, or stroke type. Disagreements were resolved through discussion until consensus was reached with the research team. Data were extracted by L.A.J.V. for the initial search and B.L.S. for the search update, and all extracted data were checked by K.v.K.

### 2.4. Quality Assessment

All included articles were scored on methodological quality using the McMaster Critical Review Form for Quantitative studies [29]. This tool was selected because all study designs were eligible for inclusion, and a cross-design appraisal approach was required. The included articles were assessed on their purpose, literature background, design, sample, outcomes, intervention, results, drop-outs, conclusions, and implications. Every item was scored as “yes” (meets criterion), “no” (does not meet criterion), or “n.a.” (not applicable). Given the predictor-focused nature of this review, item 13 (“Were the analysis methods appropriate?”) was interpreted with specific attention to the suitability of the regression methods for predicting VO_2_peak (level or change) to reflect prediction-specific threats. Quality assessment was performed by L.A.J.V. for the initial search and B.L.S. for the search update and checked by K.v.K.

## 3. Results

### 3.1. Article Selection

**Figure 1:**
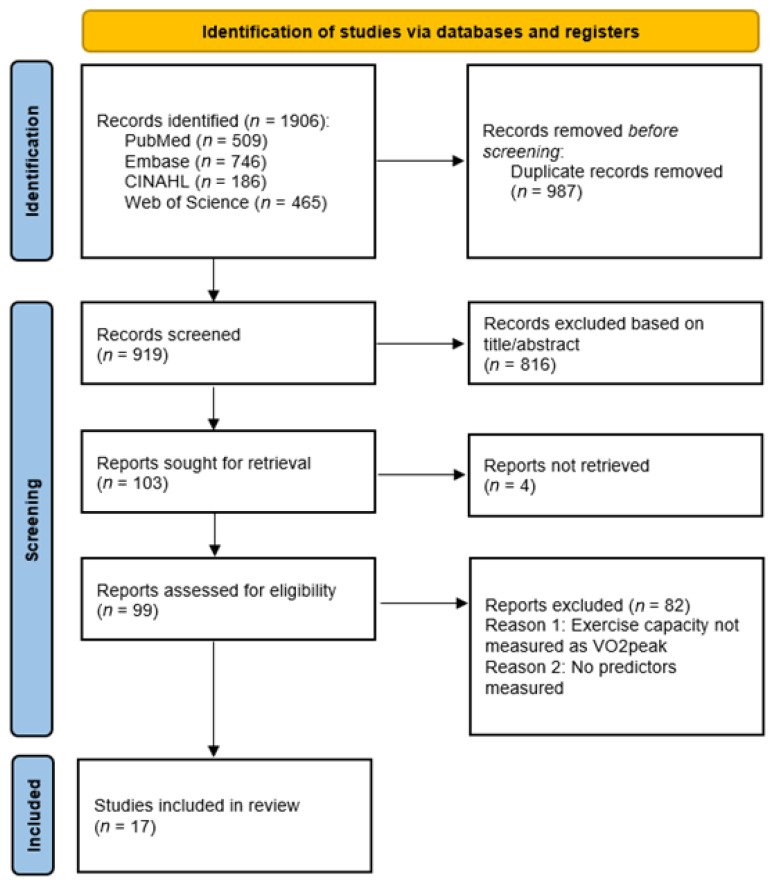
Flowchart of article selection.

The PRISMA flow diagram of the search is presented in Figure 1. After removing duplicates, the initial searches in all four electronic databases produced a total of 919 potential articles. After the title and abstract screening, a total of 103 articles were eligible for full-text screening, of which four could not be retrieved. A total of 17 articles met the eligibility criteria and were included in this systematic review. The 82 articles that were excluded during full-text screening did not meet the inclusion criteria (2) exercise capacity is measured as VO_2_peak and/or (3) predictors were measured. Reviewer agreement for title/abstract screening during the initial search was 70.9%, with a Cohen’s Kappa of 0.41 (moderate agreement). During the search update, agreement was 95.6%, with a Cohen’s Kappa of 0.48 (moderate agreement). Reviewer agreement for full-text screening was 87.4% during the initial search, with a Cohen’s Kappa of 0.62 (substantial agreement) and 87.5% with a Cohen’s Kappa 0.71 (substantial agreement) during the search update.

### 3.2. Included Articles

The extracted descriptive data from the included articles are presented in Table 1. The year of publication ranged from 2000 to 2024. The total number of participants in the included articles was 1091 (range, 11–405), with the average age ranging between 44.5 and 66.9 years. Four articles reported National Institutes of Health Stroke Scale (NIHSS), which ranged from 2.5 to 4.9, and 13 articles reported the time since stroke onset, ranging from 31 days to 5.25 years. Eleven articles reported on stroke etiology. In these articles, 609 patients survived ischemic stroke, and 174 survived hemorrhagic stroke. Eleven articles reported the side of the lesion; a total of 345 patients had a lesion on the left side, and 333 patients had a lesion on the right side.

All studies used multivariable regression models to identify predictors of exercise capacity. However, five studies reported only one significant predictor, and one study reported no significant predictors, despite including multiple variables in their models. In addition, various time points of VO_2_peak assessment were observed in relation to time since stroke onset and in relation to therapy or training. Studies also varied in their prediction targets, with 11 studies assessing absolute VO_2_peak and six studies evaluating change over time.

### 3.3. Quality Assessment

Table 2 presents the methodological quality of the 17 included papers. Although the study designs varied considerably across the included articles, cross-sectional studies were the most common (*n* = 5). In addition, four studies used a (secondary analysis of) RCT design (*n* = 4). All study designs are presented in Table 2. If applicable, all papers reported the interventions in detail, gave clinical implications and reported conclusions appropriate given the chosen statistical analysis. Five studies did not report a justification of the sample size, and six papers did not mention reasons of drop-out. Four studies did not report sufficient details on model type to ensure appropriate methodology for generating the prediction model was used (Table 2, item 13), and despite appropriate analysis, the majority of the remaining articles lacked detailed information on the final model (see Table 3).

### 3.4. Predictors of Exercise Capacity

All significant predictors are summarized in Table 3 and discussed by category (modifiable vs. non-modifiable). These results were derived from multivariable models, so the effect of each predictor may depend on other factors included in those models.

### 3.5. Modifiable Predictors of VO_2_peak Level

#### 3.5.1. Body Composition

Eight articles investigated the impact of body composition on VO_2_peak level in stroke survivors (Table 1 and Table 3). Higher BMI was a predictor of lower VO_2_peak level in four articles [31,32,40,46]. On the contrary, three articles did not find BMI to be significantly predictive of VO_2_peak level [35,39,45]. One article reported higher fat mass as a significant predictor of lower VO_2_peak level [35]. In addition, another article reported that higher lean tissue mass in both thighs was a predictor of higher VO_2_peak level, while the total lean body tissue and lean tissue in both legs were not [43].

#### 3.5.2. Lower Limb Characteristics

Three articles examined measures of muscle strength as predictors of VO_2_peak (Table 3). One study showed that stronger isokinetic knee extension strength (non-paretic leg) at three and six months post-stroke and stronger isokinetic knee flexion strength of the non-paretic leg at twelve months were significant predictors of an improved level of VO_2_peak [31]. Similarly, higher isokinetic knee extensor strength measured at 90-degree of knee flexion in the non-paretic leg significantly predicted higher VO_2_peak levels [45]. However, for the paretic leg, isokinetic knee extensor and flexor strength were not significant predictors [31]. In addition, in the same study, isometric knee extensor and flexor strength (paretic and non-paretic legs) were not significant predictors of VO_2_peak level [31]. In contrast, Kim et al. [35] identified stronger isometric extensor strength of the paretic leg as a significant predictor of an improved level of VO_2_peak, while they found that paretic flexor strength, non-paretic knee extensor and flexor strength were not significant predictors.

#### 3.5.3. Cardiorespiratory Parameters

Four articles investigated the predictive value of various cardiorespiratory parameters in stroke survivors. One study examined cardiac function as a predictor of VO_2_peak level [40], where higher Cardiac Ouput (CO) parameters and greater stroke volume were significant predictors of a higher level of VO_2_peak [40] (all models). One study examined baseline VO_2_peak as a predictor of post-intervention VO_2_peak level but showed no significance [39]. Three studies examined outcomes of a 6 min walking test as a predictor [35,40,46], with only two indicating that a longer covered distance was a significant predictor of a higher level of VO_2_peak [40,46].

#### 3.5.4. Training and Test Parameters

Moderate to vigorous physical activity was identified as a significant predictor of a higher level of VO_2_peak [37]. Baert et al. [30] reported that various other training parameters were not predictive of VO_2_peak level (Table 1). Daud et al. [34] validated a new rowing-ramp CPET test protocol, showing that higher ‘’final stage stroke power’’ was a significant predictor of a higher level of VO_2_peak, whereas mean power output and stroke rate per minute did not significantly predict VO_2_peak level.

#### 3.5.5. Other Parameters

Two studies examined other parameters as predictors for VO_2_peak in stroke survivors. Larsson et al. [37] reported that perceived fatigue was a significant predictor, with lower levels of fatigue predicting a higher level of VO_2_peak. Anxiety [37] and depression [31,37] were not found to be significant predictors of the level of VO_2_peak in stroke survivors.

### 3.6. Non-Modifiable Predictors of VO_2_peak Level

#### 3.6.1. Age and Sex

Eight articles evaluated age as a predictor of exercise capacity (Table 1). Three articles concluded that VO_2_peak level decreased significantly with age [30,32,40]. Five articles reported age as a non-significant predictor of VO_2_peak level [31,35,39,45,46].

Six articles evaluated sex as a predictor of VO_2_peak level. One article identified sex as a significant predictor of VO_2_peak level [32], with males having better exercise capacity than females. The other five articles reported sex as a non-significant predictor for VO_2_peak level in their models [30,31,35,39,40].

#### 3.6.2. Comorbidities

Four articles investigated how comorbidities predicted VO_2_peak. One article showed that the presence of diabetes was a predictor of a decreased level of VO_2_peak over 1-year, while lifestyle factors, chronic pulmonary diseases, cardiovascular diseases, and hypertension were non-significant predictors [30]. In line with this, another article did not find diabetes, coronary artery disease, hypertension, and hyperlipidemia to significantly predict the level of VO_2_peak [43]. The use of beta-blocker medication was a significant predictor of a lower VO_2_peak level in one study [32] but was non-significant in another study [46].

#### 3.6.3. Stroke-Specific Predictors

Six articles examined stroke-specific factors as predictors of VO_2_peak level. The SIS 3.0 emotion was examined and found to be a significant predictor of a decreased VO_2_peak level twelve months post-stroke in one article [31]. Side of stroke or hemiparesis was examined in three studies, with only one study showing that having right-sided hemiparesis significantly predicted the level of VO_2_peak [46]. One out of three studies examining time post-stroke as a predictor showed that a longer time post-stroke was a predictor of a lower level of VO_2_peak [32]. Higher self-selected walking velocity [43] and higher score on the Functional Ambulation Categories (FAC) at 12 months post-stroke were predictors of a higher VO_2_peak level [31], although FAC was not a significant predictor in other studies [30,32]. Three articles [30,31,40] examined other stroke-specific factors but did not find them to be significant predictors of VO_2_peak level (Table 1 and Table 3).

### 3.7. Modifiable Predictors of Change in VO_2_peak

#### 3.7.1. Body Composition

One article did not find BMI to be significantly predictive of VO_2_peak change [38].

#### 3.7.2. Lower Limb Characteristics

One article included limb characteristics in their prediction models. Lower extremity deficit and lower limb impairment, measured by the Chedoke–McMaster Stroke Assessment (CMSA), were not significant predictors of VO_2_peak change [44].

#### 3.7.3. Cardiorespiratory Parameters

Five articles investigated the predictive value of various cardiorespiratory parameters in stroke survivors. Two studies examined cardiac function as a predictor of change in VO_2_peak [33,42]. Higher Cardiac Ouput (CO) parameters were found to be significant predictors of improved VO_2_peak during the exercise test [42]. The study showed that a greater arterial-venous oxygen difference was a significant predictor of greater VO_2_peak change, while higher minute ventilation and tidal volume were not [42]. Heart Rate (HR) was not a significant predictor for VO_2_peak change during the exercise test [42] and VO_2_peak between pre- and post-intervention [33].

Three studies examined baseline VO_2_peak as a predictor of change in VO_2_peak between pre- and post-intervention. Higher baseline VO_2_peak was reported as a significant predictor of VO_2_peak measured during a follow-up measurement across two articles [38,44], while it was not significant in Macko et al. [41]. In one study, the 30 s sit-to-stand test was examined, which measures a combination of leg strength and endurance, and higher values on the test were identified as a significant predictor of greater VO_2_peak change post-intervention [33]. Two studies examined outcomes of a 6 min walk test but found no significant predictors for change in VO_2_peak [38,44].

#### 3.7.4. Training and Test Parameters

Higher exercise intensity, measured by cycling cadence during training, was a predictor of a greater increase in VO_2_peak post-intervention [38]. Macko et al. [41] indicated that an increase in velocity during gait training on a treadmill was a significant predictor of increases in VO_2_peak. Linder et al. [38], and Tang et al. [44] reported that various other training parameters were not predictive of VO_2_peak change (Table 1 and Table 3).

### 3.8. Non-Modifiable Predictors of Change in VO_2_peak

#### 3.8.1. Age and Sex

Three articles did not find age as a significant predictor of change in VO_2_peak between pre- and post-intervention [38,39,41]. Similarly, two articles reported sex as a non-significant predictor for VO_2_peak change in their models [36,38].

#### 3.8.2. Comorbidities

None of the studies evaluating predictors of VO_2_peak included comorbidities in their models.

#### 3.8.3. Stroke-Specific Predictors

Two articles [36,41] examined stroke-specific factors as predictors for change in VO_2_peak between pre- and post-intervention but did not find them to be significant predictors of VO_2_peak level or change (Table 1 and Table 3).

## 4. Discussion

This systematic review aimed to summarize predictors of exercise capacity, measured as VO_2_peak, in stroke survivors. Our findings indicate that modifiable factors such as body composition, lower limb strength, cardiorespiratory fitness (e.g., baseline VO_2_peak), training intensity and fatigue can predict exercise capacity level or change in separate prediction models in stroke patients. In addition, non-modifiable factors like age, comorbidities and stroke-specific factors were identified in separate models as significant predictors of exercise capacity level or change in stroke survivors. The heterogeneity of findings and the scarcity of studies evaluating certain predictors highlight the need for further research for confirmation and to determine one comprehensive prediction model. Nonetheless, the results highlight the potential to improve exercise capacity, and ultimately rehabilitation outcomes, through targeted interventions by considering the non-modifiable factors and addressing the modifiable factors.

### 4.1. Interpretation of the Findings

This review identified multiple modifiable predictors of VO_2_peak level or change that offer valuable insights for clinical practice. Firstly, higher training intensity, such as cycling cadence during training, more aerobic training, and higher treadmill training velocity, predicted improved exercise capacity [38,41]. Recent meta-analytic evidence in stroke populations further strengthens this observation, demonstrating that higher-intensity aerobic exercise, particularly high-intensity interval training, produces the largest improvements in VO_2_peak and cardiorespiratory fitness compared with moderate- or low-intensity exercise [47,48]. However, inconsistencies across articles suggest that the influence of training parameters is multifaceted and context-specific, highlighting the need for tailored exercise prescription in stroke rehabilitation. Additionally, isokinetic and isometric muscle strength in the lower limbs was a significant predictor of the level of VO_2_peak [31,35,45]. Although further research is needed to determine differences between paretic and non-paretic limbs, these results do indicate that targeted strengthening of leg muscles may be beneficial in rehabilitation. Nonetheless, variability related to lower limb characteristics (e.g., muscle mass and strength) may be attributed to differences in measurement methods and the heterogeneity in the specific lower limb characteristics assessed across the articles. Findings related to body composition and cardiorespiratory parameters were generally in line with existing knowledge in the general population (e.g., [49]). Especially, the cardiorespiratory parameters 6MWT and 30 s sit-to-stand test performance predicted higher VO_2_peak and might have important clinical implications. Both tests are simple, low-cost, and require minimal equipment, making them feasible for routine use in rehabilitation settings that lack access to CPET. Furthermore, higher BMI and lower cardiac output were predictors of reduced level and change in VO_2_peak in several studies, confirming the physiological relevance of these factors. However, due to variation in measurement approaches and study populations, the results were not entirely consistent across articles.

Other parameters, for example psychosocial or lifestyle related parameters, were notably underrepresented in the reviewed literature, with only two studies examining their predictive value for exercise capacity in stroke survivors. One study identified perceived fatigue as a significant predictor, with lower levels of fatigue predicting a higher level of VO_2_peak [37]. This finding suggests that subjective energy levels may play a meaningful role in aerobic performance, potentially influencing both effort tolerance and engagement in training. In contrast, anxiety and depression were not found to be significant predictors [31,37], indicating that, while these factors are important for overall recovery and quality of life [50], their direct physiological impact on exercise capacity may be limited.

For the modifiable predictors, it is important to distinguish between predictors that represent causal intervention targets and those that primarily act as markers of baseline status or disease severity. For example, baseline VO_2_peak predicting follow-up or change in VO_2_peak may reflect regression to the mean or ceiling effects rather than a modifiable mechanism. Similarly, training intensity may be confounded by mobility and functional ability, as individuals with less impairment can tolerate higher intensities. While these factors are modifiable in principle, their predictive role does not necessarily imply causality. Future research should aim to determine which modifiable predictors are markers that can be used to guide targeted rehabilitation strategies.

In terms of non-modifiable predictors, considerable heterogeneity was also observed in the significance of stroke-specific predictors of VO_2_peak level or change. This may reflect the complex nature of stroke pathophysiology [51], including variation in lesion type, location, and size among the included articles. In addition, individual physiological responses and adaptive mechanisms likely contribute to divergent findings [52,53]. Stroke affects multiple systems and results in a wide variety of impairments among individuals, which highlights the unique profiles and nature of recovery of stroke survivors [17]. This reinforces the notion that VO_2_peak in stroke survivors is shaped not only by general predictors of exercise capacity but also by neurological, functional, and psychosocial factors unique to this population.

Moreover, age was inconsistent as a predictor of VO_2_peak level or change. While some articles reported a negative association between age and the level of VO_2_peak, this relationship may be confounded by factors such as body composition, comorbidities, time since stroke and physical activity levels. For instance, older stroke survivors may experience greater muscle loss or may have more comorbid conditions that impair aerobic capacity. Furthermore, the relatively narrow age range included in the reviewed studies—reflecting the characteristics of a specific clinical population rather than the general population—may have limited the ability to detect age-related effects. This restricted variability could partly explain the inconsistent findings regarding age as a predictor of exercise capacity.

Sex did not consistently predict level or change in VO_2_peak, with only one of eight studies reporting significance. This contrasts with general-population data, where sex differences are well documented (see e.g., [49]) and suggests that stroke-specific factors may outweigh physiological differences. Future research should explore whether sex interacts with impairment severity or recovery stage to influence exercise capacity.

Finally, previous research has highlighted substantial inter-individual variability in therapy response during different phases of stroke rehabilitation [54,55,56,57], further emphasizing the need for personalized approaches.

### 4.2. Clinical Implications

Several clinical implications can be derived from our results; however, these should be interpreted with caution, as predictors were obtained from separate multivariable models, and VO_2_peak was assessed at different time points relative to time since stroke onset (acute vs. chronic) and varied in their prediction targets, with some modeling absolute level of VO_2_peak values and others predicting changes in VO_2_peak in response to an intervention. This should be taken into account when considering their application in clinical practice.

Optimizing exercise capacity in stroke survivors requires rehabilitation programs that integrate both modifiable and non-modifiable factors. Modifiable factors—such as lower limb strength, body composition, and cardiorespiratory fitness—can be effectively addressed through a combination of resistance and aerobic training. Resistance training, such as weight bearing activities, leg presses, and functional movements, can effectively improve muscular strength in stroke survivors [58,59]. Aerobic exercise, such as walking, cycling, and swimming, can enhance cardiovascular health and body composition in stroke survivors [19,20,47,60].

Although non-modifiable factors like age and stroke-specific factors cannot be changed, incorporating them into baseline assessments allows for more personalized treatment planning and aligning patients’ expectations. Evaluating broader aspects of health—such as biological age, comorbidities, and physical activity levels—further refines rehabilitation strategies and supports personalized care.

Given the complex pathophysiology of stroke and the variability in individual responses to rehabilitation, specifically in terms of VO_2_peak, a flexible and tailored approach is essential. This includes considering sex differences in long-term outcomes [61] and addressing lifestyle factors such as smoking and alcohol use, which significantly impact recovery and long-term health [62,63,64]. Promoting healthy habits post-stroke is vital, especially in light of the high recurrence rate of stroke events [65], and should be addressed as predicting factors in future research.

### 4.3. Strengths and Limitations

To our knowledge, this is the first systematic review to identify both modifiable and non-modifiable predictors of exercise capacity in stroke survivors. While previous reviews have examined the effects of exercise interventions on fitness in stroke survivors (e.g., [20,47,61]), none have systematically explored predictors of VO_2_peak level or change with a clear distinction between modifiable and non-modifiable factors. We included studies with a broad and diverse stroke population, varying in stroke type (ischemic/hemorrhagic), lesion location, and impairment severity, which enhances the generalizability of our findings. The substantial number of included studies, most with moderate to good methodological quality, further supports the reliability of the results.

However, several limitations should be noted. First, predictors were extracted from individual multivariate models reported in separate studies rather than from a unified framework. Many of the predictors were only reported as significant in a subset of studies or in one or two studies only, and variation was observed in the time point of determining VO_2_peak. Accordingly, the predictors identified cannot be interpreted as part of one comprehensive prediction model. Only predictors that were evaluated within multivariable regression models were included; univariate associations reported in the original studies were not synthesized. Second, no overarching assessment could be performed to combine predictors into a single model due to heterogeneity in the included studies. There was considerable variation in rehabilitation duration, timing of predictor and VO_2_peak measurements, and follow-up periods, which may have influenced the findings. Time since stroke onset ranged from 31 days to several years, and predictors may evolve due to spontaneous recovery or interventions. Third, heterogeneity in study designs, differences in assessment tools, definitions of predictor variables and reporting of the specification of the prediction model (e.g., regression coefficients or model performance) further complicated synthesis and interpretation. Additionally, effect sizes could not consistently be evaluated due to heterogeneous and often unstandardized reporting of coefficients in the included articles, which constrains conclusions about relative importance and clinical relevance beyond statistical significance. These methodological limitations were not fully captured in the quality assessment. Although the McMaster Critical Review Form enabled consistent assessment of quality across heterogeneous designs, it is not a prediction-specific risk-of-bias tool. As a result, evaluating prognostic and prediction-model studies and capturing modeling-specific sources of bias were limited in the quality assessment. Because of design heterogeneity, implementing a single prediction-specific tool for all included articles was not feasible. Nonetheless, we adapted item 13 of the McMaster tool to reflect modeling considerations and acknowledge that residual prediction-model–specific limitations remain.

### 4.4. Future Research

Addressing the identified limitations in future research is essential to advance understanding of predictors of exercise capacity in stroke survivors. Standardized measurement methods and consistent reporting of predictors are needed to improve comparability across studies. Longitudinal research can clarify how changes in modifiable and non-modifiable factors influence exercise capacity and recovery over time in one comprehensive model to predict both level and change in VO_2_peak and reveal interactions that support personalized rehabilitation strategies.

Further investigation into how factors such as age or stroke severity but also lifestyle factors affect the impact of strength and endurance training may guide personalized interventions. Integrating exercise and dietary strategies to improve lifestyle habits should be prioritized to enhance long-term outcomes. Psychological factors remain underexplored in the context of VO_2_peak prediction; only two studies assessed these, with fatigue emerging as a potential predictor. Systematic exploration of mental and emotional influences on rehabilitation engagement and physical performance is warranted.

## 5. Conclusions

In conclusion, this systematic review identified key modifiable (such as BMI, fat mass, lower limb strength, cardiorespiratory fitness (e.g., baseline VO_2_peak), training intensity and perceived fatigue) and non-modifiable (such as age, diabetes, side of the stroke and time since stroke onset) predictors of level and change in exercise capacity in stroke survivors, based on separate prediction models. Focusing on the modifiable and non-modifiable factors in rehabilitation programs has the potential to significantly enhance exercise capacity. However, this review also highlights the complexity of understanding consistent predictors of exercise capacity, given the influence of the multifaceted nature of stroke pathophysiology and inter-individual variability. Future research is needed to develop a comprehensive prediction model.

## Figures and Tables

**Table 1:** Study characteristics.

Reference	Study Design	Sample Size (M/W)	Age (M ± SD)	Measures of Physical Capacity (Time Point)	Intervention	Time Since Stroke Onset and/or Stroke Severity	Stroke Type and/or Localization	Evaluated Predicting Factors of Physical Capacity
Baert [30]	Descriptive; Longitudinal	33 (23/10)	59.0 ± 11.3	A symptom- limited ramp exercise test; VO_2_peak (3, 6 and 12 months post-stroke)	NA	NIHSS 4.5 ± 4.0	Ischemic (26) Hemorrhagic (7) Left (17) Right (10) Both sides (5)	Age, sex, pre-stroke Baecke work, sport and leisure index (BPAQ), smoking habits, diabetes, chronic pulmonary diseases, cardiovascular diseases, overweight, hypertension, stroke type, stroke area, side of lesion, NIHSS, Mini-Mental State Examination (MMSE), Trunk Impairment Scale (TIS), Barthel Index (BI), Functional Ambulation Categories (FAC), Rivermead Motor Assessment (RMA-GF), length of stay at the stroke rehabilitation unit, time between stroke onset and admission to the stroke rehabilitation unit, number of patients returning home at 3, 6, and 12 months post-stroke, therapy received
Baert [31]	Descriptive; Longitudinal	40 (25/14)	57.2 ± 11.4	A symptom- limited ramp exercise test; VO_2_peak (intake rehabilitation, and 3, 6 and 12 months post-stroke)	NA	NIHSS 4.9 ± 4.2	Ischemic (31) Hemorrhagic (9) Left (19) Right (13) Both sides (7)	Age, sex, BMI, log four skinfolds, waist circumference, knee extensor/flexion strength (paretic, non-paretic limb, isometric, and isokinetic), Beck Depression Inventory-II (BDI-II), RMAGF, FAC, BI, Nottingham Extended Activities of Daily Living (NEADL), 10 MWT, Stroke Impact Scale 3.0 (SIS), Modified Ranking Scale (MRS), time spent in physical therapy/occupational therapy/sports
Blokland [32]	Retrospective; cohort	405 (284/121)	63 ± 16.5	CPET; VO_2_peak (during rehabilitation treatment)	NA	31 ± 55.5 days post-stroke	Ischemic (340) Hemorrhagic (61) Left (204) Right (164) Other (37)	Age, sex, beta-blocker medication, BMI, side of stroke, type of stroke, first/recurrent stroke, time since stroke, motricity index, FAC, Berg Balance Scale (BBS), Utrecht Scale of Evaluation of Rehabilitation mobility sub-score (USERm)
Bosch [33]	Pilot feasibility study	17 (11/6)	HIIT: 59.5 ± 16.4 Control: 55.4 ± 11.9	GXT; VO_2_peak (change pre-post intervention)	High intensity interval training (HIIT) or no intervention (control)	HIIT: 24.5 ± 14.6 Control: 29.8 ± 5.6 months post-stroke	Ischemic (13) Hemorrhagic (4) Left (6) Right (11)	Change scores (post-pre) for peak Heart Rate (HR), self-selected walking speed, 30 s sit-to-stand (STS), peak rate of perceived exertion (RPE)
Daud [34]	Experimental	11 (6/5)	44.5 ± 24.04	CPET; VO_2_peak (>3 months post-stroke)	NA	> 3 months post-stroke	Ischemic (2) Hemorrhagic (9) Left (2) Right (9)	Final stage stroke power, power output, stroke rate (spm)
Kim [35]	Retrospective; observational; cohort	83 (47/36)	62.95 ± 13.9	ETT; VO_2_peak (baseline before rehabilitation treatment)	NA	32.54 ± 24.61 days post-stroke	Ischemic (59) Hemorrhagic (24) Left (32) Right (51)	Age, sex, BMI, muscle mass, fat mass, paretic/non-paretic knee extensor/flexor strength, six meter walk distance (6 MWD), 10 m walk velocity (10 MWV), BBS (Korean version)
Lam [36]	Post hoc analysis of two RCTs	52 (34/18)	66.8 ± 1.1	T-EX; VO_2_peak (change pre-post intervention; relative to baseline)	Treadmill exercise	NIHSS: 4.08 ± 0.35	Ischemic (52) Right-sided (20)	Age, sex, stroke therapy interval (months), stroke location, lesion volume, NIHSS, trial (Germany vs. US)
Larsson [37]	Cross-sectional	74 (47/27)	64 ± 13	CPET (treadmill); VO_2_peak (>3 months post-stroke)	NA	~3 months after stroke; mRS: 1.1 ± 0.8	Right (28) Left (23) Bilateral/(right-)posterior (12)	Moderate to vigorous physical activity (based on accelerometer data), step count, perceived fatigue, anxiety, depression
Linder [38]	Secondary analysis of data from 2 RCTs	FE: 16 (12/4) VE: 15 (10/5) Control: (12/1)	FE: 51 ± 12 VE: 60 ± 14 Control: 58 ± 11	CPET; VO_2_peak (change pre-post intervention)	Forced aerobic exercise + repetitive task practice (FE), or voluntary exercise + repetitive task practice (VE) or non-aerobic exercise (control)	FE: 12 VE: 16 Control: 12 months post-stroke	Chronic stroke	Age, sex, BMI, group allocation, baseline 6 MWD, baseline VO_2_peak, aerobic exercise intensity (HRR percentage), exercise rate (cycling cadence), power
Linder [39]	Secondary analysis of data from an RCT	FE: 25 (15/10) RTP: 25 (14/11)	FE: 60.1 ± 12 RTP: 59.6 ± 9.6	CPET; VO_2_peak (post-intervention)	Forced rate aerobic exercise + repetitive task practice (RTP) or RTP only	FE: 18 RTP: 23 months post-stroke	FE: Ischemic (20) Hemorrhagic (5) RTP: Ischemic (18) Hemorrhagic (7)	Age, sex, BMI, beta-blocker usage at baseline, baseline VO_2_peak.
Liu [40]	Cross-sectional	59 (52/7)	50.0 ± 11.7	CPET; VO_2_peak (during treatment)	NA	NIHSS: 2.5 ± 1.8 <3: (12), 3–6 (29), 7–12 (18) months post-stroke	Cerebral infarction (38) Cerebral hemorrhage (21) Left (26) Right (33)	Age, sex, BMI, time after stroke, 6 MWD, HRpeak, Stroke Volume (SV) peak, Cardiac Output (CO)peak, HRend, SVend, COend
Macko [41]	RCT	61 (44/18)	T-AEX: 63 ± 10 Control: 64 ± 8	Peak exercise test; VO_2_peak (change pre-post intervention)	Treadmill aerobic exercise (T-AEX) or care as usual with low-intensity walking (control)	T-AEX: 35 ± 29 Control: 39 ± 59 months post-stroke	Chronic hemiparetic gait after ischemic stroke	Age, latency since stroke, initial VO_2_peak, gait deficit severity, treadmill training velocity
Oyake [42]	Cross-sectional; observational	18 (14/4)	60.1 ± 9.4	Symptom-limited graded exercise test; VO_2_peak (during exercise test, change rest vs. peak exercise)	NA	67.1 ± 30.8 days post-stroke	Ischemic (11) Hemorrhage (7) Left (10) Right (8)	Tidal volume, minute ventilation, heart rate, cardiac output, arterial-venous oxygen difference
Ryan [43]	Cross-sectional	26 (22/4)	66.9 ± 9	Maximal effort graded testing; VO_2_peak (chronic stroke)	NA	3.2 ± 4.7 years post-stroke	Index cerebral infarction	Body lean tissue, lean tissue of both legs and thighs, coronary artery disease, hypertension, diabetes, hyperlipidemia, smoking history, self-selected walking velocity
Tang [44]	Secondary analysis of data from a prospective cohort study	Response: 15 (11/4) Non-response: 17 (11/6)	Response: 63.7 ± 12.1 Non-response: 61.7 ± 13.3	Symptom-limited graded maximal exercise test; VO_2_peak (change pre-post intervention)	Responders or non-responders to aerobic + resistance training program	Response: 34.7 ± 32.1 months post-stroke Non-response: 23.8 ± 19.3	N.R.	Chedoke–McMaster Stroke Assessment (CMSA), lower limb impairment, BBS, 6 MWT, baseline VO_2_peak, exercise time, exercise intensity
Wang [45]	Descriptive; cross-sectional	35 (24/11)	57.10 ± 12.14	Maximal tolerance test; VO_2_peak (chronic stroke)	NA	1.84 ± 1.86 years post-stroke	Infarctic (17) Hemorrhagic (18) Left (19) Right (16)	Age, BMI, duration, Functional Independence Score (FIM), 90-degree torque of paretic/non-paretic leg
Woodward [46]	Cross-sectional; observational	53 (31/22)	59.0 ± 8.8	GXT; VO_2_peak (chronic stroke)	NA	64.0 ± 86.0 months post-stroke	Left (25) Right (28)	Age, beta-blocker medication, peak treadmill speeds, steps/day, resting rate, 6 MWT-fastest velocity (FV) distance, side of hemiparesis, BMI

Cardiopulmonary exercise test (CPET); National Institutes of Health Stroke Scale (NIHSS); High Intensity Interval Training (HIIT); Maximal treadmill graded exercise test (GXT); symptom-limited exercise tolerance test (ETT); Peak-effort T-EX test on a treadmill (T-EX); forced aerobic exercise and upper extremity repetitive task practice (FE); voluntary aerobic exercise and upper extremity repetitive task practice (VE); Repetitive task practice (RTP); treadmill aerobic training (T-AEX); Randomized Controlled Trial (RCT); Modified Rankin Scale (mRS). NA = not applicable.

**Table 2:** Detailed methodological quality assessment of the included studies following McMasters Critical Review Form for Quantitative studies.

Reference	1	2	3	4	5	6	7	8	9	10	11	12	13	14	15	16
Baert [30]	y	y	Descriptive, longitudinal	33	n	y	y	y	na	na	na	y	y	y	na	y
Baert [31]	y	y	Descriptive, longitudinal	40	n	y	y	y	na	na	na	y	y	y	na	y
Blokland [32]	y	y	Retrospective cohort study	405	n	y	y	y	na	na	na	y	y	y	na	y
Bosch [33]	y	y	Pilot feasibility study	15	n	y	y	y	y	na	na	y	y	y	n	y
Daud [34]	y	y	Single case design with test–retest approach	11	y	y	y	y	y	na	na	y	y	y	n	y
Kim [35]	y	y	Retrospective observational cohort study	83	n	y	y	y	na	na	na	y	y	y	n	y
Lam [36]	y	y	Post hoc analysis of 2 RCTs	52	n	y	y	y	y	y	y	y	y	y	n	y
Larsson [37]	y	y	Cross-sectional study	74	y	n	y	y	na	na	na	y	y	y	y	y
Linder [38]	y	n	Secondary analysis of 2 RCTs	44	n	y	y	y	y	na	na	y	n	y	n	y
Linder [39]	y	y	Secondary analysis of 1 RCT	50	y	n	y	y	y	y	y	y	n	y	y	y
Liu [40]	y	y	Cross-sectional study	59	y	y	y	y	na	na	na	y	y	y	na	y
Macko [41]	y	y	RCT	61	n	y	y	y	y	y	y	y	n	y	y	y
Oyake [42]	y	y	Cross-sectional observational design	18	y	n	y	y	na	na	na	y	y	y	na	y
Ryan [43]	y	y	Observational correlational study	26	n	n	y	y	na	na	na	y	y	y	na	y
Tang [44]	y	y	Secondary analysis of data from a prospective cohort study	32	n	y	y	y	na	na	na	y	y	y	n	y
Wang [45]	y	y	Descriptive, cross-sectional	35	n	n	y	y	na	na	na	y	n	y	na	y
Woodward [46]	y	y	Cross-sectional observational design	53	n	y	y	y	na	na	na	y	y	y	na	y

1: Was the purpose stated clearly? 2: Was relevant background literature reviewed? 3: What was the design of the study? 4: What was the sample size of the study? 5: Was the sample described in detail? 6: Was the sample size justified? 7: Were the outcome measures of the aerobic test reliable for the specific study population (if not described, assume no)? 8: Were the outcome measures of the aerobic test valid for the specific study population (if not described, assume no)? 9: Was the intervention described in detail? 10: Was contamination avoided? 11: Was co-intervention avoided? 12: Were results reported in terms of statistical significance? 13: Were the analysis methods appropriate? 14: Was clinical importance reported? 15: Were drop-outs reported? 16: Were conclusions appropriate given the study methods? na: not applicable.

**Table 3:** Significant predictors of exercise capacity.

Reference	Dependent Variable	Model Type	Significant Predictors of Exercise Capacity	F	Coefficient/(Unstandardized)Beta (SE)	CI (95%)	Standardized Beta	*p*-Value
**Predictors of VO_2_peak level**								
Baert [30]	VO_2_peak (mL/kg/min)	Multivariate mixed model with backward selection	Time x age	14.26	NR	NR	NR	<0.001
			Time x diabetes	11.31	NR	NR	NR	<0.001
Baert [31]	VO_2_peak (mL/kg/min)	Linear regression with backward selection	Model 1: at 3 months					
			Isokinetic knee extension strength (non-paretic leg) (Nm)	20.82	NR	NR	NR	<0.001
			Model 2: at 6 months					
			Isokinetic knee extension strength (non-paretic leg) (Nm)	38.28	NR	NR	NR	<0.001
			Model 3: at 12 months					
			Isokinetic knee flexion strength (non-paretic leg) (Nm)	64.46	NR	NR	NR	<0.001
			Functional Ambulation Categories (FAC) (score)	19.87	NR	NR	NR	<0.001
			Body Mass Index (BMI) (kg/m^2^)	8.14	NR	NR	NR	<0.001
			Stroke Impact Scale (SIS) 3.0 emotion (score)	9.49	NR	NR	NR	<0.001
Blokland [32]	VO_2_peak (mL/kg/min)	Multiple linear regression with backward selection	Age (years)	NR	0.994 ^B^	0.991–0.996	NR	<0.001
			Sex (men/women)	NR	0.865 ^B^	0.822–0.911	NR	<0.001
			Beta-blocker medication (no/yes)	NR	0.875 ^B^	0.830–0.924	NR	<0.001
			BMI (kg/m^2^)	NR	0.984 ^B^	0.978–0.991	NR	<0.001
			Time since stroke (TSI) (d)	NR	0.936 ^B^	0.873–0.946	NR	<0.001
			USERm (score)	NR	1.008 ^B^	1.005–1.011	NR	<0.001
Daud [34]	VO_2_peak (mL/kg/min)	Stepwise linear regression	Final stage stroke power	25.32	0.232 ^B^	0.128–0.336	0.859	0.001
Kim [35]	VO_2_peak (mL/kg/min)	Multivariate linear regression with backward selection	Paretic isometric extensor strength (Nm)	NR	NR	NR	NR	<0.001
			Fat mass (kg)	NR	NR	NR	NR	0.005
			10 min Walking Velocity (m/s)	NR	NR	NR	NR	<0.001
Larsson [37]	VO_2_peak/kg (%)	Stepwise backward linear regression	Fatigue (score)	NR	−3.88 ^B^	−6.36; −1.60	−0.31	0.004
			Moderate to vigorous physical activity (min/day)	NR	0.38 ^B^	0.15; 0.63	0.4	0.002
Linder [39]	VO_2_peak (mL/kg/min)	Multivariable linear regression	No significant predictors					
Liu [40]	VO_2_peak (mL/kg/min)	Stepwise backward multiple linear regression	Model 2:					
			SVpeak (mL)	NR	−0.242 ^C^	NR	−0.685	NR
			COpeak (L/min)	NR	2.076 ^C^	NR	1.303	NR
			Model 3:					
			BMI (kg/m^2^)	NR	0.196 ^C^	NR	0.167	NR
			Age (years)	NR	−0.060 ^C^	NR	−0.223	NR
			6 MWD (m)	NR	0.010 ^C^	NR	0.398	NR
			Svend (mL)	NR	−0.416 ^C^	NR	−1.158	NR
			COend (L/min)	NR	3.587 ^C^	NR	1.402	NR
			Model 3a:					
			6 MWD (m)	NR	0.009 ^C^	NR	0.383	NR
			Svend (mL)	NR	−0.364 ^C^	NR	−1.015	NR
			COend (L/min)	NR	3.465 ^C^	NR	1.355	NR
Ryan [43]	VO_2_peak (mL/kg/min)	Stepwise multiple regression	Model	NR	NR	NR	NR	<0.001
			Lean tissue mass (both thighs) (kg)	NR	NR	NR	NR	NR
			Self-selected walking velocity (m/s)	NR	NR	NR	NR	NR
Wang [45]	VO_2_peak (L/min)	Linear regression analyses	90 DT NPL (Nm)	NR	0.559 ^C^	NR	NR	<0.05
Woodward [46]	VO_2_peak (mL/kg/min)	Stepwise multiple linear regression	Model	8.23	NR	NR		<0.001
			6 MWT-FV distance (m)	NR	0.02 ^C^	NR	NR	<0.05
			Right hemiparesis	NR	−4.45 ^C^	NR	NR	<0.05
			BMI (kg/m^2^)	NR	−0.35 ^C^	NR	NR	<0.05
**Predictors of change in VO_2_peak**								
Bosch [33]	Change in VO_2_peak (mL/kg/min)	Backward stepwise linear regression	30 s sit-to-stand performance (repetitions)	15.69	NR	NR	NR	0.004
Lam [36]	Change in VO_2_peak (mL/kg/min)	General linear models with forward enter procedure	Trial sample (United States)	NR	NR	NR	NR	0.0312
Linder [38]	Change in VO_2_peak (mL/kg/min)	Multivariate linear regression	Overall model	2.66		NR	NR	0.03
			Cycling cadence (>60 revolutions per/minute)	NR	NR	NR	NR	0.01
			Baseline VO_2_peak (mL/kg/min)	NR	NR	NR	NR	0.02
			VE group allocation	NR	NR	NR	NR	0.02
Macko [41]	Change in VO_2_peak (mL/kg/min)	Regression analyses	Treadmill training velocity (m/s)	NR	NR	NR	NR	<0.05
Oyake [42]	Change in VO_2_ from rest to peak exercise (mL/min)	Stepwise multiple regression analysis	Arterial-venous oxygen difference (mL/100 mL)	NR	83.85 ^C^ (8.54)	NR	0.665	<0.001
			Cardiac output (L/min)	NR	152.22 ^C^ (16.22)	NR	0.636	<0.001
Tang [44]	Change in VO_2_peak (%)	Multivariate regression analysis with backward procedure	Model	3.48	NR	NR		0.045
			Baseline VO_2_peak (mL/kg/min)	NR	−1.82 ^B^ (0.70)	NR	−0.47	0.02
			Berg Balance Scale (score)	NR	0.70 ^B^ (0.40)	NR	0.38	0.06

Utrecht Scale of Evaluation of Rehabilitation mobility sub-score (USERm); Voluntary Aerobic Exercise (VE); 6 Minute Walking Distance (6 MWD); Stroke Volume (SV); Cardiac Output (CO); 90-degree torque of non-paretic leg (90 DT NPL); 6 Minute Walk Test Fastest Velocity (6 MWT-FV). ^C^ = Coefficient; ^B^ = Beta. The table is structured in two sections, distinguished by header rows in bold with a grey background: (1) predictors of VO_2_peak level, and (2) predictors of VO_2_peak change.

## Data Availability

No new data were created or analyzed in this study. Data sharing is not applicable to this article.

## Supplementary Materials

The following supporting information can be downloaded at: https://www.mdpi.com/article/10.3390/healthcare14030382/s1.
