# Supplementary material for: Modifiable and Non-Modifiable Predictors of Exercise Capacity in Stroke Survivors: A Systematic Review
Source: Healthcare (Basel). 2026 Feb 3;14(3):382. doi: 10.3390/healthcare14030382 (PMC12897659; doi:10.3390/healthcare14030382)
Supplement: Supplementary file 1 [file healthcare-14-00382-s001.zip › healthcare-4080087-supplementary.pdf]

1. **PubMed**

(‘’Stroke’’[Mesh] OR stroke[ti] OR poststroke[ti] OR cva[ti] OR cerebrovascular*[ti] OR "cerebral vascular*"[ti])

AND

(predict*[tiab] OR determinant*[tiab] OR factor*[tiab] OR aspect*[tiab] OR component*[tiab] OR influence*[tiab] OR parameter*[tiab])

AND

("‘’physical fitness’’"[Mesh] OR "exercise capacity"[tiab] OR "exercise tolerance"[tiab] OR "endurance capacity"[tiab] OR "physical capacity"[tiab] OR "physical endurance"[tiab] OR "cardiorespiratory fitness"[tiab] OR VO2[tiab] OR "oxygen uptake"[tiab] OR "aerobic capacity"[tiab])

2. **Embase**

(Stroke/exp OR stroke:ti OR poststroke:ti OR cva:ti OR cerebrovascular*:ti OR 'cerebral vascular*':ti)

AND

(predict*:ti,ab,kw OR determinant*:ti,ab,kw OR factor*:ti,ab,kw OR aspect*:ti,ab,kw OR component*:ti,ab,kw OR influence*:ti,ab,kw OR parameter*:ti,ab,kw)

AND

('physical fitness’/exp OR 'exercise capacity':ti,ab,kw OR 'exercise tolerance':ti,ab,kw OR 'endurance capacity':ti,ab,kw OR 'physical capacity':ti,ab,kw OR 'physical endurance':ti,ab,kw OR 'cardiorespiratory fitness':ti,ab,kw OR VO2*:ti,ab,kw OR 'oxygen uptake':ti,ab,kw OR 'aerobic capacity':ti,ab,kw)

NOT

‘conference abstract’/it

3. **CINAHL EBSCO**

((MH ‘’Stroke’’+) OR (TI stroke) OR (TI poststroke) OR (TI cva) OR (TI cerebrovascular*) OR (TI "cerebral vascular*"))

AND

((TI predict* OR AB predict*) OR (TI determinant* OR AB determinant*) OR (TI factor* OR AB factor*) OR (TI aspect* OR AB aspect*) OR (TI component* OR AB component*) OR (TI influence* OR AB influence*) OR (TI parameter* OR AB parameter*))

AND

((MH "‘’physical fitness’’+") OR (TI "exercise capacity" OR AB "exercise capacity") OR (TI "exercise tolerance" OR AB "exercise tolerance") OR (TI "endurance capacity" OR AB "endurance capacity") OR (TI "physical capacity" OR AB "physical capacity") OR (TI "physical endurance" OR AB "physical endurance") OR (TI "cardiorespiratory fitness" OR AB "cardiorespiratory fitness") OR (TI VO2* OR AB VO2*) OR (TI "oxygen uptake" OR AB "oxygen uptake") OR (TI "aerobic capacity" OR AB "aerobic capacity"))

4. **Web of Science**

TI=(“stroke"’ OR “poststroke” OR “cva” OR “cerebrovascular*” OR “cerebral vascular*”)

AND

TS=(predict* OR determinant* OR factor* OR aspect* OR component* OR influence* OR parameter*)

AND

TS=(“physical fitness” OR “exercise capacity” OR “exercise tolerance” OR “endurance capacity” OR “physical capacity” OR “physical endurance” OR “cardiorespiratory fitness” OR “VO2*” OR “oxygen uptake” OR “aerobic capacity”)
